# Psychological factors and production behaviors of Chinese undergraduate EFL learners

**DOI:** 10.1371/journal.pone.0288014

**Published:** 2023-07-10

**Authors:** Zheng Wang

**Affiliations:** Xiamen University Tan Kah Kee College, China; Zhejiang University, CHINA

## Abstract

Psychological factor have received much attention in education research. The present mixed-methods study focuses on the effect of foreign language enjoyment (FLE), foreign language classroom anxiety (FLCA) on production behaviors among 182 Chinese English as a foreign language (EFL) learners in FL teaching classes. The major findings are as follows: (1) Chinese university students prefer written production behaviors to oral ones, and personal or pair-work oral practice to onstage oral demonstrations due to foreign language classroom anxiety (FLCA); (2) gender does not affect foreign language enjoyment (FLE), foreign language classroom anxiety (FLCA), or production behaviors; (3) language competence or test scores did not directly affect students’ decisions to speak English or not; (4) Team cooperation, classroom atmosphere, attitude towards English, and interesting materials mediated FLE and FLCA, thus affecting the students’ readiness for language output or production behaviors. Of these above-mentioned variables, team cooperation and classroom atmosphere are two most important factors in enhancing positive emotion and production behaviors. The implications of the study are to help teachers optimize their classroom activities o harness the emotions of learners, boost their FLE and lower their FLCA, and improve their willingness to speak a foreign language.

## Introduction

The limited improvements in learners’ oral English ability have made it imperative for English language educators in China to develop a new pedagogy more oriented to Chinese educational practices. In light of this, Wen [1–3] proposed the production-oriented approach (the POA), one that integrates the strengths of Western instruction approaches into Chinese contexts to help improve English learners speaking and writing abilities at higher education institutions. With the POA, teachers arrange productive activities for English learners who often suffer from anxiety and are unwilling to communicate. The purpose of the POA is to encourage students to communicate in English or to better accomplish productive activities while input serves as an enabler of productive activities.

The design and research of POA are similar to the western research of “willingness to communicate”. Recent research has proved the significant role of learners’ positive and negative emotions in foreign language classrooms [4, 5, 6]. Among the long list of emotions, the combination of Foreign Language Enjoyment (FLE) and Foreign Language (Classroom) Anxiety (FLCA) has caught the most scholarly attention. FLE and FLCA have been widely recognized as prevalent among language learners across the world [7, 8, 9, 10, 11, 12, 13].

Previous research has also found that Chinese foreign language learners suffer higher levels of FLCA compared to international samples. We assume that FLCA may be a key factor impeding student’s production initiative. As is shown in many research, affective factors such as motivation, attitude, and anxiety, boredom have a direct impact on foreign language acquisition [4, 7, 8, 14, 15, 16, 17]. Considering this mainstream interest in emotional and psychological factors in second language acquisition, it is not just appropriate but also necessary to evaluate the link between POA and emotional and psychological factors. To fill this gap, this research aims to explore how production behaviors and outcomes are related to such psychological factors as FLE and FLCA and the role of different variables in positive emotion and production behaviors.

With Chinese second-year undergraduates as participants, the present research aims to answer the following questions:

1. What are the features of foreign language production-oriented behaviors of Chinese college students? How do psychological factors correlate to the features of production behaviors?
2. What is the correlation between students’ 1) FLE, 2) FLCA, and 3) willingness or preferences for language output, and between language output and students’ foreign language competence?
3. Does gender affect FLE, FLCA, and their production behaviors?
4. To what extent do learner-internal and learner-external variables boost positive emotion and predict Chinese learners’ production behaviors in English? What are the pedagogical and psychological implications for identifying students’ preferences and variables in the POA?

## Literature review

### POA research in the Chinese and foreign contexts

The POA is a newly-developed teaching method prevalent in mainstream foreign language teaching classrooms in Mainland China.

In the past few decades, the pedagogical methods used in mainstream education in Mainland China were text-centered and input-based. That is to say, English instruction takes the text as an end rather than a means, and input-processing is the dominant learning task. Such English pedagogy can at most enhance students’ communication skills. Studies have also noted that Chinese students, as well as other Asian students, are described as typically quiet in class, suffering from anxiety to communicate [18]. There has existed in China the phenomenon of ’Dumb English’ for many years. The POA was therefore been developed as a potential remedy for students’ unwillingness to speak English and poor spoken English skills. This is because when students are not willing to communicate and try out productive tasks, English language education programs essentially fail.

According to the POA, the ultimate objective of college students’ English learning should be to develop their productive skills and use English for communication. The three teaching procedures of POA—motivating, enabling, and assessing—are put into practice effectively and successfully in Chinese foreign language learning contexts [1–3, 19]. Among these three teaching procedures, the second enabling phase plays a more vital role than the first and third phases.

The three teaching hypotheses of the POA—“output-driven”, “input-enabled”, and “selective learning”—serve as a theoretical foundation for carrying out this approach. These hypotheses propose that to realize “output-driven” hypotheses, teachers should carefully select, for the assigned output tasks (speaking or writing), reading or listening as input materials. Such materials become “output-driven” enablers leading students to better outcomes [20]. In other words, receptive or input activities, such as listening and reading, must provide students with relevant ideas, sentence patterns, linguistic expressions, and grammar structures, all of which serve as selective language input enablers of better language output.

As an innovative pedagogy integrating the strengths of Western instruction approaches with Chinese characteristics, the POA has been developed mainly at universities in Mainland China. In recent years, a small number of researchers outside China interested in this pedagogy have already expressed their opinion and raise related issues about POA, such as famous scholars Paul Kei Matsuda [21] and Henry Widdowson [22]. As a well-articulated set of principles and guidelines that systematically incorporate various ideas, POA makes sense as a way of helping students become active language users who can go beyond just understanding or absorbing information produced by other people. However, so far the research about POA pedagogy has never been fully conducted in other countries. Novel and unfamiliar to western researchers as it is, POA is a potential remedy for poor spoken English dilemma among FLE learner in China. It is an innovative teach pedagogy worth to be noted and can be well applied to teaching contexts where students’ unwillingness to speak English is a prevalent issue.

Although the POA as a teaching method has succeeded brilliantly in improving Chinese students’ spoken English, academia home and abroad has never fully explored the relationship between psychological factors of learners’ and their production behaviors. These factors are complexly interconnected and play a vital role in motivating learners’ willingness to communicate [23, 24].

Based on the above, this research proposes a novel perspective of designing an optimal way of organizing students’ productive activities in which students’ psychological states are fully considered. To be specific, we aim to find out the most appropriate design of production activity to promote positive emotion and better language production behaviors.

### Studies on FLE and FLCA

Since emotional and psychological studies were introduced to the field of foreign language learning, many researchers have begun to explore both negative and positive psychological states as influencing factors in foreign language learning [9, 10, 13, 15, 25, 26].

Positive emotion is one of the factors that can play a role in the process of second/foreign language (FL) learning [27–31]. Research studies on FLE have focused on different aspects of FLE, such as its conceptualization, measurement, antecedents, and correlation in the language learning process [32, 33]. Reviewing the existing literature indicated that FLE can lead to better academic achievement [33, 34], L2 motivation [9], and social-behavioral learning engagement [35].

The most noticeable research on the positive psychological aspects is the foreign language enjoyment (FLE) scale. This standard scale has 21 items with Likert scale ratings designed by Dewaele and MacIntyre [8], reflecting positive emotions toward the language learning experience. In China, however, Foreign Language Enjoyment (FLE) has caught the most scholarly attention. For instance, Li et al. [32] conducted the first systematic survey of FLE in Chinese foreign language learning contexts. They developed the Chinese foreign language enjoyment scale (CFLES) with three dimensions: personal experiences, teachers’ attitudes, and classroom atmosphere and activities. Their qualitative analysis of students’ descriptions also unfolded that an individual student’s experience of FLE originated from a wide range of learner-internal and learner-external variables, such as the learner’s self-realization, the teacher, the peer group, and the classroom environment. In addition, Jin and Zhang [33] investigated factors underlying FLE among 320 EFL high school students. The most enjoyable FLC episodes, as Falout [36] qualitatively revealed, are classroom activities that allow some degree of autonomy, though spatial factors in the classroom could contribute to a positive atmosphere.

As one of the main sources of negative psychological states, anxiety has been extensively investigated over the past several decades. Horwitz [8], for example, developed the Foreign Language Classroom Anxiety Scale (FLCAS), which consists of 33 items in relation to communication apprehension, fear of negative evaluation, and test anxiety. The findings that FLCA had a debilitating effect on foreign language learning and achievement have been confirmed by many studies in different countries all over the world [32]. An analysis of the qualitative data revealed that low scores on English tests and fear of teacher criticism were major sources of FLCA [37].

IF FLCA had a debilitating effect on foreign language learning and achievement, can FLCA also impede production behaviors or students’ willingness to speak English? There is, however, scarce research combining FLE and FLCA with students’ foreign language production activities.

### Studies on the relationships between the POA and psychological aspects

In the past decade, psychological aspects in foreign language learning and teaching, such as FLCA and FLE and their impacts on foreign language learning, have attracted increasing attention from researchers. The existing literature has also demonstrated that FLE and FLCA are linked to a range of learner-internal and classroom-specific variables [35, 38]. Meanwhile, other studies have explored the potential roles of different variables, such as age, teacher friendliness, and learners’ experience of FLE and FLCA [8, 9, 28, 38].

None of these findings, however, have explored the relations between the POA and the psychological and emotional aspects of students. Because emotions are closely associated with language output behaviors, the POA teaching system might be optimized if we consider the psychological factors of learners as motives [39].

This study aims to create, from an innovative perspective, a learner-friendly classroom that increases students’ FLE and decreases students’ FLCA. It designs a more appropriate teaching method as enabling factors to better facilitate students’ productive activities. Among the three POA processes of motivating, enabling, and assessing, the second is the key to successful teaching, therefore worthy of the most attention. The carefully selected reading or listening materials become input enablers towards better production outcomes. Thus, in addition to preparing materials relevant to the assigned speaking or writing tasks, the careful planning of preferred production approaches and class activities can also serve as another enabling factor for better production outcomes.

As society advocates systematically understanding the psychological traits of foreign language learners, the present study aims to delve into this area and encourage more active production outcomes by designing an optimal way of organizing production activities.

## Methodology

### Participants

Participants in this study were second-year undergraduates from an ordinary university in China, where the Chinese government requires undergraduates to take college English courses compulsively. I adopted self-selection sampling which entailed putting out a call for volunteer students and forming a sample group from those who willingly participated in the research. I intentionally selected the second-year students because they have had a whole year of EFL learning experience at university. Of the 182 participants, there were 122 male students (67%) and 60 female students (33%). They all spoke and learned Chinese (local dialect and Mandarin) as their L1, and English as their L2. All data were valid. In addition, verbal consent has been obtained from all student participants.

### English competence

IELTS (the International English Language Testing System) was used as a standard model test to measure participants’ actual English achievement. As a task-based test covering the four language skills (listening, reading, writing, and speaking), IELTS provides accurate rubrics for assessing participants’ language abilities. According to the test results, most participants scored between 4 and 5 points, which means most participants’ English level is between pre-intermediate and intermediate levels

### Instruments

In China, the emotions that foreign language learners experience in the classroom may be different from those in other parts of the world [40]. Based on CFLES [32] and FLCAS [41], I designed a new version for the CFLES and the FLCAS, respectively, to explore non-English majors’ emotions related to their preferences for language output models and teaching organization in EFL classrooms.

The first part of my CFLES included questions about students’ personal information: their name, gender, and the grade they were in. The second part was the 21-item FLE scale. The 21 internally consistent items were rigorously translated and paraphrased based on Dewaele and MacIntyre’s (2014) CFLES, with five-point Likert scales rating (1 = strongly disagree, 2 = disagree, 3 = neutral, 4 = agree, 5 = strongly agree).

Likewise, I extracted most of the 33 items in my FLCAS from Dewaele and MacIntyre’s (2014) FLCAS after deleting and revising some items not fit for Chinese contexts. These items were used to assess learners’ anxiety levels and were based on five-point Likert scales (1 = strongly agree, 2 = agree, 3 = neutral, 4 = disagree, 5 = strongly disagree). The 33 questions were divided into four dimensions: lack of confidence, nervousness, fear of public speaking, and fear of poor performance.

In this study, Cronbach’s alpha for the FLE scale was 0.703 and KMO for the FLE scale was 0.851 while Cronbach’s alpha for the FLCA was 0.902 and KMO was 0.800. Confirmatory factor analysis confirmed the validity of the modified version for the FEL and FLCA scales.

The third section of the survey was a self-made *Questionnaire on the Attitudes to the Production-oriented Approach*. The questionnaire included three parts: selecting production methods, POA classroom organization, and open-ended subjective questions. The Likert scale was made according to three dimensions of students’ choice of foreign language production, classroom organization, and oral practice. As regards the open-ended questions, participants were asked to describe one most enjoyable AND one anxious language output experience in the EFL class.

### Data collection and analysis

Questionnaires were distributed to students through Tencent Instant Messenger (QQ platform). Data was collected through “Wenjuanxing” (https://www.wjx.cn), a professional online questionnaire survey platform. Valid questionnaires were 182, of which 71.2% were from science and engineering students, and 28.8% from economics and management ones.

The participants were verbally informed of the purpose and nature of the survey as well as their rights to participate in or withdraw from the survey at the beginning of the questionnaires. Considering the correlational research design in the present study, participants were asked to provide their names in the questionnaire but were assured that their names would not appear in publications. In other words, we had to match their data for the variables based on their name. The quantitative data were analyzed by SPSS25.0, to measure participants’ attitudes towards POA teaching mode, levels of FLE, and FLCA, *effect of variables on POA*, whereas the qualitative data were analyzed with NVivo 12 to display the typical profile of students’ psychological states and the different factors influencing their language production.

## Results

### Attitude towards POA pedagogy

The first research question examined the features of non-English majors’ production behaviors. And a single-sample t-test was performed to explore students’ different attitudes towards oral and written production.

Table 1 shows that the “POA attitude” scores (Mean value = 3.55) of Chinese undergraduates were significantly higher than 3 (the median) and lower than 4, indicating that they are positive about using the POA. There were significant differences between written and oral production. More specifically, attitudes towards written behaviors (Mean Value = 3.77) scored much higher than oral (Mean Value = 3.33), suggesting that Chinese students are more positive about the former. The result echoes with previous finding that Chinese students are more proficient in written English than in oral English due to FLCA. We hope to find out the psychological factors accounting for the difference.

**Table 1:** Attitude towards the POA.

		Mean Value	Standard Deviation	Minimum Value	Maximum Value
Primary Indicator	Secondary Indicator				
production behaviors		3.55	0.82	2	5
	Written Production	3.77	0.89	2	5
	Oral Production	3.33	1.08	1	5

### Preferences analysis of the production behaviors

We surveyed some college English teachers, who in foreign language classrooms have most frequently adopted six English production behaviors—topic discussion, oral quiz question, role-play dialogue, translation of phrases or sentences, compound dictation, and short written report. For these six behaviors, ANOVA for repeated measurement was used to conduct a significance test. The results showed that the difference is extremely significant (F = 5.925, p<0.001) (see Table 2). According to further pairwise comparisons, the scores of the six production behaviors are the translation of phrases or sentences > compound dictation sentences or word phrases > short-answer written report > oral quiz questions > topic discussion activity > role-play interview and dialogue. There was, however, no significant difference in the selection of the three oral practice approaches. The results suggested that students prefer the traditional written-oriented production behaviors to the oral, in that the latter may trigger FLCA. As expected, this data analysis further confirmed the first finding.

**Table 2:** Preferences analysis of the production behaviors.

	Mean value	Standard Deviation	F	P	Post Hoc Test
Topic discussion	3.29	1.042	5.925	0.000	4>5>6>2>1>3
Oral quiz question	3.31	0.971			
Role-play dialogue	3.28	1.087			
Translation of phrases or sentence	3.71	0.821			
Compound dictation	3.57	0.915			
Short written report	3.56	0.888			

(Note: 1. Topic discussion; 2. Oral quiz question; 3. Role-play dialogue; 4. Translation of phrases or sentences; 5. Compound dictation; 6. Short written report)

### Preferences analysis of oral practice organization modes

To find out how the organization mode of output task can affect FLE and FLCA, ANOVA for repeated measurement was used to conduct the significance test in FLE for the classroom organization in the oral English practice class, including four main oral production behaviors: students’ self-practice, paired oral practice, group discussion, and face-all oral practice. Post hoc Gabriel tests showed significant differences between the attitudes towards these four types of oral practices (F = 10.869, p<0.001) (see Table 3), and further pairwise comparisons showed the scores of the four: students’ self-practice > paired oral practice > group discussion > face-all oral practice. The oral practice mode gave them the most enjoyment in personal self-practice in their seats, while public oral English presentation triggered the highest level of anxiety. This result was in line with the results of interviews and questionnaires that followed.

**Table 3:** Preferences analysis of oral practice organization modes.

	Mean Value	Standard Deviation	F	P	Post Hoc Test
Students’ self-practice	3.78	0.868	10.869	0.000	1>2>3>4
Paired oral practice	3.52	0.971			
Group discussion	3.45	0.976			
Face-all oral practice	3.14	1.148			

(Note: 1. Students’ self-practice; 2. Paired oral practice; 3. Group discussion; 4. Face-all oral practice)

### Gender difference in FLE/FLCA and production behaviors

Table 4 shows the independent-sample t-test on the gender differences in students’ written and oral production preferences. As can be seen from the table, the two genders had negligible differences in choosing “written” or “oral” (p>0.05).

**Table 4:** Analysis results of T-test.

	1.0 (*n* = 93)	2.0 (*n* = 63)		
preferences for Written Production	2.18±0.92	2.33±0.82	-1.046	0.297
preferences for Oral Production	2.55±1.09	2.89±1.03	-1.956	0.052

* p<0.05 ** p<0.01

Table 5 shows that different gender samples had no salient differences in the enjoyment and anxiety (p>0.05), revealing that gender does not affect FLCA, FLE, or preferences for written or oral production.

**Table 5:** Analysis results of T-test.

	1.0 (n = 93)	2.0 (*n* = 63)		
FLE	49.34±8.99	49.10±7.50	0.181	0.856
FLCA	70.70±19.34	70.57±18.50	0.041	0.967

* p<0.05 ** p<0.01

This finding is contrary to the general belief that female students have more interest and FLE than male ones in speaking a foreign language.

### Pearson correlations between the POA, test scores, and FLE/FLCA

To identify the significant relationships between students’ production behaviors and FLE/FLCA, the Pearson correlation analysis was used to reveal the correlation (see Table 6) which showed that students’ production behaviors were positively correlated with enjoyment, negatively correlated with foreign language anxiety, and had little influence on test scores.

**Table 6:** Pearson correlations between the POA, test scores, and FLE/FLCA.

	FLE
Production	0.450**
	FLCA
Production	-0.268**
	Test scores
Production	0.054

### Effect of variables on the POA

The subsequent research focuses on the effects of learners’ classroom emotions on the POA since willingness to participate in production activities is also influenced by personal characteristics and teaching contexts [42]. These two constantly changing factors are simultaneously shaped by learner-internal and learner-external variables in FL classrooms. Thus, to what extent these variables predict Chinese learners’ POA and positive emotion?.

In the follow-up research, participants were first asked about their attitude toward English on a 5-point Likert-type scale. Responses ranged from “very unfavorable (1)”, “unfavorable (2)”, “neutral (3)”, “favorable (4)”, and “very favorable (5)”. The same descriptors were used to determine attitudes toward the teaching material and modes. Then they were asked about their team cooperation spirit and classroom atmosphere, with responses ranging from “very negative (1)”, “negative (2)”, “neutral (3)”, “positive (4)”, and “very positive (5)”. To avoid any ambiguity, I have to acquire an accurate definition of classroom social atmosphere from a reliable source: Classroom or learning climate refers to the “intellectual, social, emotional, and physical environments in which our students learn.” [43]. The last question focused on the students’ degree of autonomy in class, with responses ranging from “hardly ever (1)”, “not very often (2)”, “sometimes (3)”, “usually (4)”, to “all the time (5)”.

Pearson correlation analyses(see Table 7) with a Bonferroni correction (p < .001) showed that the 6 independent variables were significantly positively linked to the POA (see Table 7). Further analysis will permit the calculation of the exact amount of variance that each independent variable explains their willingness to communicate.

**Table 7:** Pearson correlation analyses between independent variables and the POA.

Independent variables	POA
Attitudes towards English	.419
Attitudes towards teaching modes	.536
Attitudes towards teaching materials	.321
Positive classroom atmosphere	.651
Team cooperation between classmates	.727
Degree of autonomy	.248

**means P<0.01

AS this study aims to design a more appropriate teaching method to regulate FLE and FLCA in order to better facilitate students’ productive activities, the next step is to find out different effects of these variables on classroom emotion and POA. A stepwise multiple regression analysis(see Table 8) was conducted to test whether the six independent variables significantly predicted participants’ attitudes towards the POA. A significant regression equation analysis was made to obtain the result, where 2 out of 6 variables predicted about 45% variance. The strongest positive predictor was “team cooperation”, accounting for 49% (β = 0.49, p < .0001). Other positive predictors were “classroom atmosphere” which explained an additional 40% of the variance (β = 0.40, p < .0001) (see Fig 4), followed by “attitude towards English” and “teaching materials”. Moreover, the teaching mode and degree of autonomy were at no unique variance or had not much effect on the production behaviors of Chinese undergraduates.

**Table 8:** Multi-regression analysis of independent variables.

	Nonstandard		Standard coefficient	t	Sig.
	B	deviation	β		
	0.059	0.324		3.869	0.007
Attitudes towards English	0.230	0.088	0.194	2.613	0.010
Attitudes towards teaching mode	0.122	0.085	0.119	1.429	0.155
Attitudes towards teaching materials	0.166	0.086	0.132	1.926	0.056
Positive classroom atmosphere	0.403	0.090	0.398	4.481	0.000
Team cooperation among classmates	0.498	0.088	0.478	5.679	0.000
Degree of autonomy	0.118	0.078	0.097	1.504	0.135
R²	0.606				
F	38.211				

Dependent variable: the POA

All these results suggested that the POA is affected by a range of learner-internal and learner-external variables, some of which can regulate classroom emotion and atmosphere and play a vital role in students’ decisions to speak or not.

### Scatter figure

**Fig 1:**
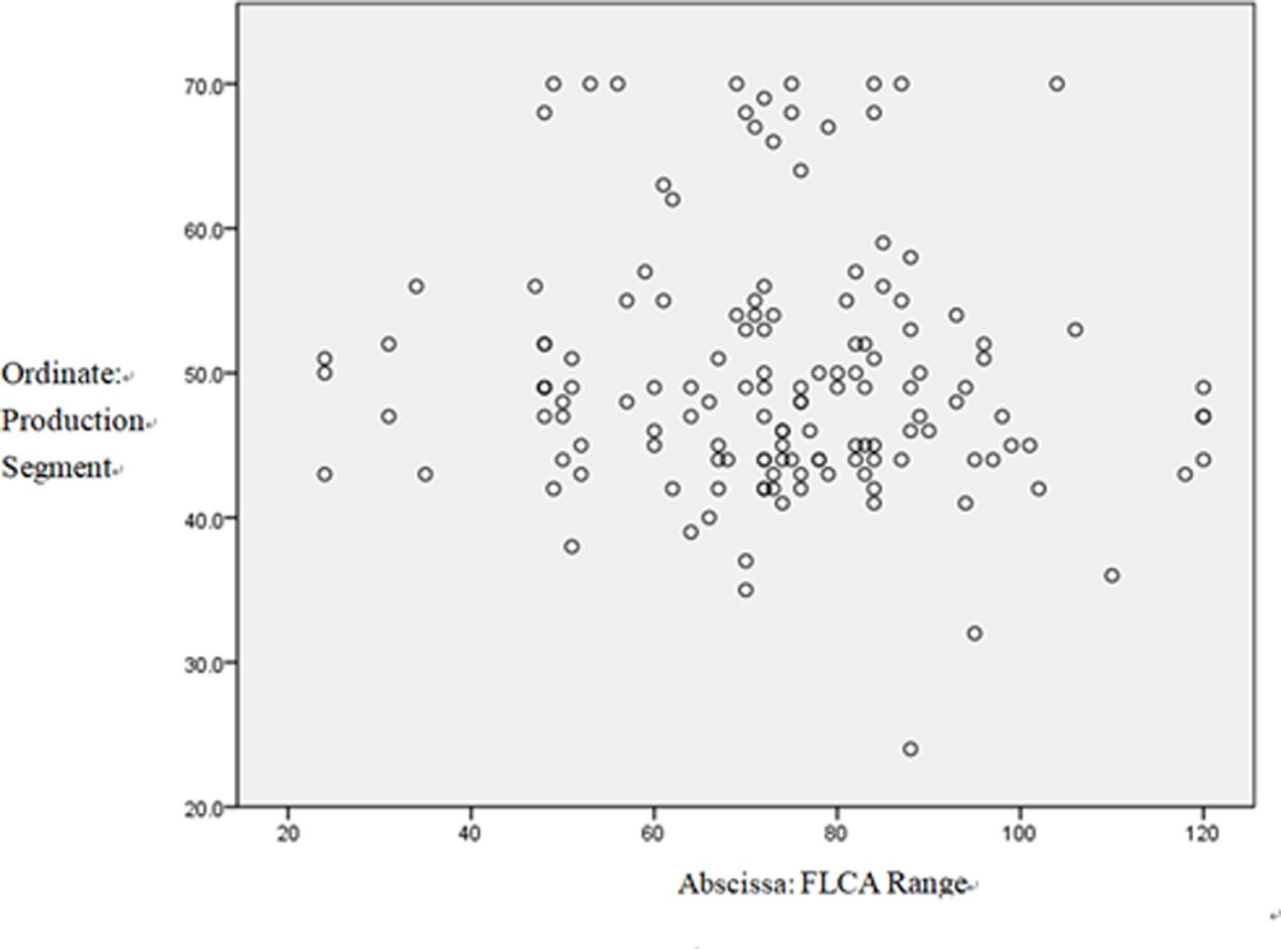
The effect of FLCA on students’ production behaviors.

**Fig 2:**
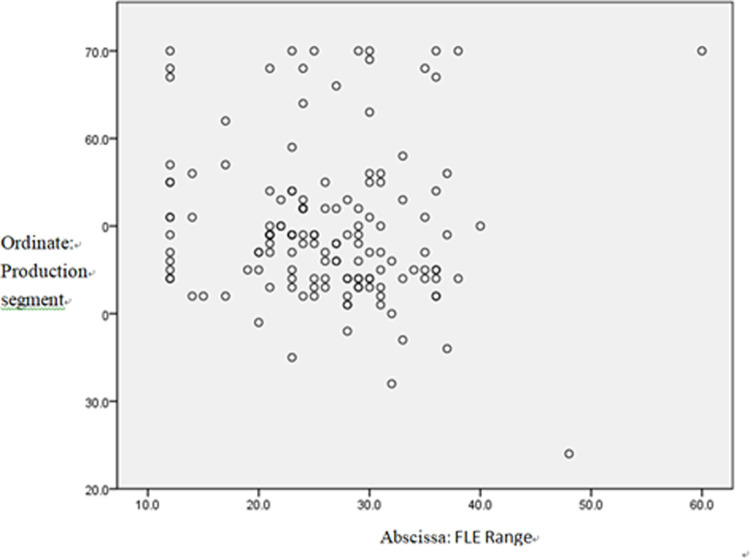
The effect of FLE on students’ production behaviors.

**Fig 3:**
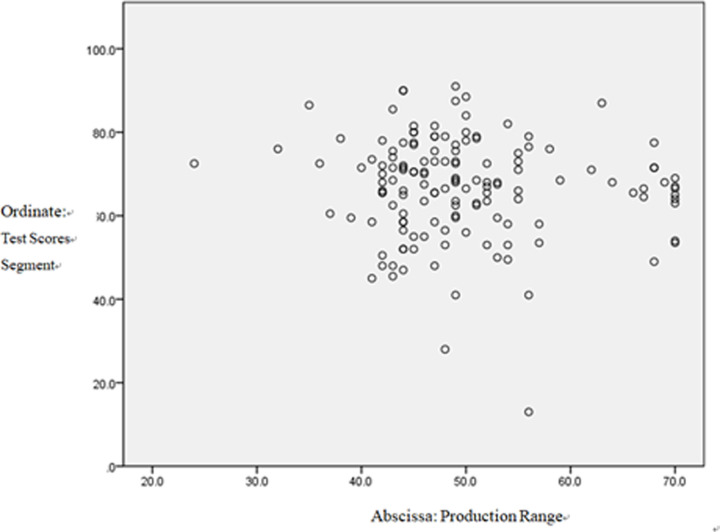
The effect of students’ production behaviors on test scores.

Figs 1–3 present the distribution quantity and related frequency distribution of students’ production attitudes, enjoyment, and anxiety. Combined with the Pearson correlation analysis, most data in Fig 1 are distributed below the diagonal line, which indicates a negative correlation on the whole.

Most data in Fig 2 are distributed above the diagonal line. Therefore the data are generally positively correlated.

In Fig 3, On the whole, therefore, the data are evenly distributed on both sides of the diagonal line with discrete features.

Low anxiety corresponds to more active production behaviors, meaning that students are more willing to speak or write English; high anxiety corresponds to less positive production behaviors. On the contrary, higher levels of enjoyment induce more active production behaviors, ones that will not be affected by test scores.

### Qualitative analysis of open-ended questions

Further, the qualitative information from the questionnaire was analyzed, “to explore the deeper meanings to add interpretive depth and breadth to the analysis” [10]. The question settings are to describe one most enjoyable AND one anxious language output experience, hoping to find the factors causing FLE and FLCA.

**Fig 4:**
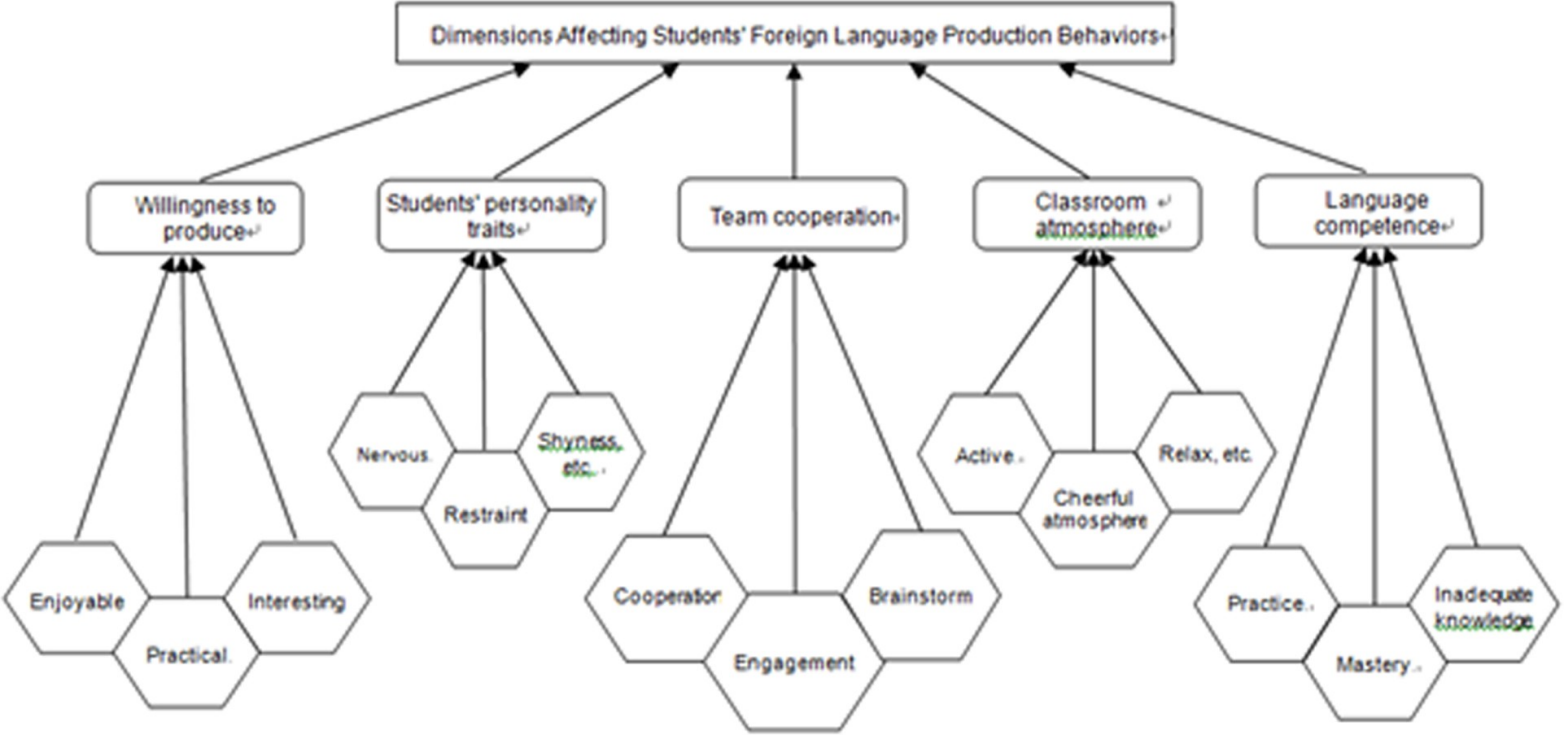
The major dimensions affecting students’ production behaviors.

A qualitative questionnaire survey presents the underlying reasons why the students in the English class are reluctant to speak English and prefer written production. The descriptive statistics were calculated to display the typical profile of students’ psychological states and the different factors influencing their language production. The data were analyzed by NVivo 12 to identify the key dimensions affecting the production behaviors. A close analysis of participants’ responses to the open-ended questions generated the following dimensions: students’ personality (24.9%), classroom atmosphere (20.4%), language competence/knowledge reserve (18.8%), team spirit and cooperation (18.3), and willingness to speak or write (17.6%). (see Fig 4)

### FLCA—Students’ introversion (see Fig 4)

In the dimension of students’ personality, most participants mentioned that they were afraid of speaking English in front of the teacher and their classmates. Several mentioned personality traits—such as shyness, restraint, and nervousness as a factor jeopardizing their output. Their views are fully represented in extracts 1 to 3.

#### Extract 1

Off-stage practice. I do not want to perform in front of the whole class. I prefer practicing oral English on my own.

#### Extract 2

I feel very embarrassed and nervous when speaking English. I am afraid of losing face.

#### Extract 3

I have a social phobia. I do not like speaking English in public. I feel shy and restrained and cannot express myself clearly in English.

#### FLE—Classroom Atmosphere and Team Cooperation (see Fig 4)

Classroom atmosphere and team cooperation are reported by students as positive motivating factors affecting their output behavior

#### Extract 4

The novel, interesting, and meaningful classroom activities, such as role-play, interviewing, and watching class-related videos, led to effective learning.

#### Extract 5

Pair work or group discussion can lighten the classroom atmosphere and group members share interesting stories, thus promoting peer relationships.

**Other factors** (see Fig 4)

#### Extract 6

Limited vocabulary prevents me to express myself freely.

My pronunciation is not standard and I cannot utter a single complete sentence in English.

#### Extract 7

I do not like memorizing English words. I tend to forget new words unless I mark them down.

#### Extract 8

I do not deem English very important to my future career development. I learn English in order to pass the exams. I am not truly interested in practicing English in class.

Based on the questionnaire findings, we can conclude that the production behaviors are most often affected by personality and classroom atmosphere, contrary to the general belief that language incompetence is the major obstacle for students practicing oral English.

## Discussion

The present study provides a more in-depth understanding of the effect of psychological factors on learners’ language production behaviors and the relationship between the POA, FLE, and FLCA.

### Learners’ attitudes and preferences for production behaviors

The first research question examined the features of foreign language production-oriented behaviors of Chinese college students. The attitude scores (Mean value = 3.55) from the POA questionnaire of Chinese undergraduates are significantly higher than the median 3. This result shows that after the POA was introduced by foreign language authorities, the POA has in recent years achieved initial results because most students have taken a positive attitude towards it. They reported feelings of freedom from tension when completing written production tasks, which echoes the findings reported in previous POA studies.

Previous studies have also found that Asian students, including Chinese students, are typically quiet in class and unwilling to speak English. They have better English reading and writing skill. This may be because Asian students tend to be more reserved and introverted than western ones. My open-question research also showed that when speaking a foreign language, China’s English learners experience a high level of anxiety because of their personality traits; they suffer greater anxiety to speak than to write in English.

The result of paired sample t-test analyses further confirmed that attitudes towards the three written production behaviors scored significantly higher than those of the oral. Due to FLCA and diffidence, most students prefer written production behaviors and the traditional oral practice mode such as oral quiz questions and group discussion. To this end, this research compares multi-practice or production approaches, for instance, students self-practice, pair work, group work, and face-all oral presentations. The research results reveal and answer the first research question that students’ preferred personal oral-English practice in their seats, a way that gave them the most enjoyment. Following this way were pair work and group discussion. The production or practice mode they most disliked was the face-all oral English presentation when they experienced the highest level of anxiety. The above results were further confirmed by a qualitative questionnaire survey where many students revealed that they were unwilling to try out oral production practice or in-class face-all presentations because of such psychological factors as shyness, nervousness, restraint, and the feeling of losing face. Under the framework of positive psychology, designing an optimal way of organizing students’ production activities may lead to more FLE and more active production behaviors [30, 43].

### POA–FLE and FLCA–Test score correlations

In terms of the second research question, Pearson correlation analysis revealed a perfect negative significant correlation between FLCA and the POA but a positive one between FLE and POA.

Similar to previous studies, anxiety can not only be a debilitating factor in learning a foreign language, but dampen students’ initiative to process, write, or speak a foreign language. Previous studies have also found that a high level of FLE allows students to absorb the foreign language better, improve foreign language learning, and erase the after-effects of negative emotions, all of which can be hugely beneficial to FL teachers and learners [4, 14]. Likewise, this study also suggests that FLE can lead to more positive production attitudes and bring about better production outcomes, particularly regarding comfort levels of speaking a foreign language.

Unsurprisingly, students who were more positive towards production-oriented behaviors reported both significantly more FLE and less FLCA. Surprisingly, the attitude towards production was unrelated to test scores, suggesting that test scores do not directly affect production behaviors. As is shown in Scatter Diagram 2, the production behaviors only showed a limited effect on students’ test scores.

As is revealed by the qualitative research, language competence was only ranked the third contributing factor in production-oriented behaviors, preceded by the personality and classroom atmosphere factors. The scatter plot graph showed that the students’ data were distributed on the upper sides of the diagonal, which indicates that students with better test scores may be a little more active in production behaviors.

As regards the scatter plot graph, the data distribution in the three scatter diagrams showed discrete features. In the whole diagram, the variation of the points showed significant individual differences in students’ FLE, FLCA, and POA attitudes. This reveals that the level of FLE, FLCA, and attitudes towards the POA vary greatly among individual students and that their production behaviors constantly fluctuate. Hence, it is surprising to find that students’ language competence does not affect their production behaviors.

### Gender roles in FLE, FLCA, and production behaviors

The third research question dealt with the gender roles in FLE, FLCA, and production behaviors. Previous studies examining the role of gender in FL learners’ experience of FLE and FLCA in other countries yielded inconsistent results. Dewaele and MacIntyre [8] investigated potential gender differences, revealing that female students experience more enjoyment in foreign language lessons. Likewise, Dewaele and MacIntyre [44] confirmed females’ tendency to experience higher levels of FLCA. Other studies, however, have presented contrary results: students who enroll in English classes exhibit the same levels of FLE regardless of their genders [38].

So far, only a limited number of studies have examined the role of gender in learners’ POA production experience. No single study has yet been conducted in Chinese contexts, where traditionally female foreign-language majors outnumber male ones and are thought to have better language competence. The present study is the first gender-role report on FLE, FLCA, and the POA. The results of this study indicated no significant difference in levels of FLE between male and female learners (t = 0.181, p = 0.856). This finding is contrary to the general belief that female students have more interest than male ones in a foreign language. Similarly, for the two genders, there was no significant difference between levels of FLCA (t = 0.041, p = 0.967).

As is shown in Table 5, there was also no stark gender difference in the participants’ preferences for written or oral production. Although females are considered more willing to communicate than males, the two genders saw the same contour for written AND oral production.

### Predictive effect (Multi-regression) of variables linked to production behaviors

The result of pearson correlation analysis reveal that students’ language competence does not affect their production behaviors. We made the assumption that there must be other crucial factors influencing their decisions to speak or not.

Previous research on the contributing factors to FLE has rather unanimously indicated that teacher-related factors play a more important role than learner-related factors in FLE. In this respect, teacher variables such as emotional support, use of humor, level of friendliness, respect toward students, tone of voice, and positive mood were found to influence learners’ FLE. The multi-regression analysis is to identify the independent variables linked to production behaviors, variables that can help teachers create the optimal environment for learner emotions and therefore boost their willingness to speak and, by extension their foreign language learning.

In the regression models, the strongest positive predictors of the POA were team cooperation and classroom atmosphere, a result further confirmed by my qualitative analysis. This suggests that team cooperation plays the most fundamental role in boosting positive emotions of learners and predicting the POA. Novel though this finding is, it fits in East-West cultural differences of placing importance on the group or individuals. As is shown by previous research, a good classroom atmosphere is a vital component of teaching and learning that helps to establish and maintain rapport between teacher and students, builds motivation and confidence among the learners, and facilitates the processes of teaching and learning [25, 38]. As is shown by this finding, classroom atmosphere also significantly predicted students’ engagement as well as production behaviors.

Insignificantly correlated with production behaviors are the variables of teaching modes and the degree of student autonomy. The former turned out not to predict production outcome, which means changing teaching modes will not affect production behaviors; the latter will not guarantee more active production behaviors, as disengaged students typically enjoy the autonomy to keep silent.

## Conclusion

This study examined the interplay among FLE, FLCA, and production behavior among Chinese EFL learners. The results revealed that FLE had a significant positive impact on learners’ production behaviors while FLCA had a debilitating effect on them. Moreover, it found that the design of appropriate or preferred organization modes of production tasks may affect learners’ production outcomes through the mediation of emotion. However, Language competence or test scores did not directly affect students’ decisions to speak English or not. Having underscored the significance of creating a positive and enjoyable learning environment, the author also finds that team cooperation, classroom atmosphere, attitude towards English, and interesting materials mediated FLE and FLCA, thus affecting the students’ readiness for language output or production behaviors. Of these above-mentioned variables, team cooperation and classroom atmosphere are the two most important factors in enhancing positive emotion and production behaviors.

The first research result showed that Chinese students have better written English ability than oral. That is why POA teaching methods aims to encourage students to speak English for communication and to further improve students’ oral English ability. My qualitative results of the open-ended questionnaire revealed that personality traits and FLCA of Chinese students are two major factors contributing to the choice of written-oriented production behaviors. Written production, however, may serve as an effective enabling factor for the preparation of speaking English, which means teachers can help students write down or translate what they want to express as a means of mitigating students’ speaking anxiety.

Secondly, the innovative perspective of this paper is that it explores an optimal way of organizing students’ production activities as an attempt to encourage more active production behaviors. This paper also demonstrates that good pedagogical practices are crucial to maintaining and boosting students’ motivation levels and positive emotions. For example, instead of face-all presentations when students feel the highest level of anxiety, teachers may arrange self-practice or pair work to release students’ negative emotions, such as anxiety, tension, and restraint.

The open-ended question revealed that students regard speaking poor English in public as a shame of losing face and damaging self-esteem. Thus if they do not have to perform on the podium, they can better attend to, process, and speak a foreign language. And if teachers deem on-the-podium practice necessary, they should choose volunteers or give them sufficient preparation time, one or two weeks, for example. Furthermore, students should also be encouraged to use Powerpoint slides or written materials as a mediator or enabling factor to guide their speech or oral practice.

The findings can be used to inform future research on the variables that influence EFL learners’ FLE, FLCA and production behaviors. The study suggests that educators and course designers should focus on creating a positive and enjoyable learning environment, optimizing the practice mode and output task design, and motivating learners’ positive emotions as a way to enhance their willingness to speak English. Therefore, educators should consider incorporating these strategies and variables into their language output task design to foster a more effective and engaging learning experience for EFL students.

This research will help expand the scope of previous research on the POA, FLCA, and FLE. Nevertheless, three limitations to this exploratory study should be acknowledged. First, although the tables and figures in this paper were tested robust enough to reflect general meaningful tendencies, the samples were selected from a single undergraduate university, which may reduce the generalizability of the results. Future research may address this limitation by collecting data from diverse sources. Second, the cross-sectional design limited the validity of the data analyses. Future research may longitudinally delve into the relationships among these variables. Third, the complexity of psychological factors hinders the straightforwardness to determine the category of a particular method.
